# Spatiotemporal variations of vegetation and its determinants in the National Key Ecological Function Area on Loess Plateau between 2000 and 2015

**DOI:** 10.1002/ece3.5165

**Published:** 2019-04-15

**Authors:** Haiguang Hao, Yuanyuan Li, Huiyuan Zhang, Ruixue Zhai, Haiyan Liu

**Affiliations:** ^1^ State Key Laboratory of Environmental Criteria and Risk Assessment Chinese Research Academy of Environmental Sciences Beijing China

**Keywords:** climatic factors, ecological compensation policy, Loess Plateau, National Key Ecological Function Area, Normalized Difference Vegetation Index, stepwise regression analysis

## Abstract

China defined 25 National Key Ecological Function Areas in 2010 and adopted various measures to support ecosystem restoration in these areas. During the process of environment policymaking, it is important to observe the variation of vegetation and its driving factors. In this paper, we chose the National Key Ecological Function Area (NKEFA) on Loess Plateau as the study area. Based on MODIS‐NDVI data between 2000 and 2015, the trend analysis was used to depict the change in NDVI and the stepwise regression analysis method was used to quantitatively assess its determinants. The results show that: (a) The vegetation coverage in study area was low in the northwest and high in the southeast, corresponding to the distribution of precipitation and temperature. (b) NDVI in the growing season increased remarkably from 0.2841 in 2000 to 0.4199 in 2015 with a linear tendency of 0.085/10a. About 71.22% of the study area experienced an extremely significant increasing of NDVI, while only 0.03% of the total area suffered from significant decreasing of NDVI. (c) Compared to climatic factors, ecosystem conservation policies, and labor transfer contributed more to the vegetation changes in the study area. In order to ensure ecological security and sustainable development in these areas, it is necessary to maintain the continuity of ecological compensation policy. Moreover, developing targeted eco‐compensation policies and encouraging farmers to participate in nonfarm employment are effective ways to reach a win–win outcome of reducing the ecosystem pressure and improving the welfare of rural households.

## 1 INTRODUCTION

The spatiotemporal variation of vegetation is the result of interaction between climate factors and human activities. At large scale, human activities usually refer to human disturbances, such as cultivation activities, road traffic, and urban land use (Gao et al., 2017). But in National Key Ecological Functional Areas (NKEFA) on Loess Plateau, human activities like ecological protection policies or labor force transfer may have more influence on vegetation condition.

The Major Function Oriented Zoning issued by the State Council of China in 2010 identified 25 NKEFA. On the one hand, these areas as national ecological security safeguard are targeted to provide ecological products and ecosystem services. On the other hand, NKEFA mostly overlapped with poverty‐stricken areas, faced with serious ecological degradation under a continuously extensive economic growth model (Li, Yuan, Gao, & Xu, 2014). By 2015, the government had arranged 251.3 billion yuan for these regions, to strengthen ecological protection and improve the economic level.

Severe ecosystem degradation threatens regional sustainable development and ecological security in NKEFA on Loess Plateau. Since 1999, the Chinese government has implemented many ecological protection policies in this area, such as the Grain for Green (GFG) project, Natural Enclosing and Prohibiting Grazing project, Natural Forest Conservation project, Ecological Public‐welfare Forests Protection policy, Grassland Subsidy policy, and transferring payment for NKEFA. Many studies have shown a significant improvement in local ecosystem after the implementation of ecological protection policies, indicating that these policies had received good results (Su, Peng, Xie, & Huang, 2011), particularly in fragile areas (Ouyang et al., 2016). Since the late 1980s, the net primary productivity (NPP) decreased in South China due to urban expansion, but North China experienced an increase in NPP with the help of vegetation restoration program, especially on Loess Plateau (Li et al., 2018).

Except for ecological protection policies, labor transfer also influences on vegetation change. During the process of urbanization, a large number of rural labors in mountainous areas emigrated to urban areas, searching for more job opportunities and higher salaries, which bring about an impact on vegetation greenness (Li, Li, Tan, & Wang, 2017). More people moved to urban areas, engaging in nonagricultural jobs, which reduced the pressure on local ecosystem, especially in places where people mainly depended on agriculture and animal husbandry for their livelihood. Li et al. (2016) found that the emigration of agricultural labor had significantly improved vegetation cover in Inner Mongolia, which means compared to natural factors, agricultural labor affects more on vegetation cover.

As for the climatic factors impacting the vegetation change, a similar conclusion has been drawn in different researches. That is the correlation between vegetation and climatic factors was higher in monthly scale than yearly scale on Loess Plateau (Bai, Bai, & Wang, 2014; Duo, Zhao, Qu, Jing, & Xiong, 2016; Zhang, Fang, & Shi, 2016). More researches focused on comparing the impact of human activities and climatic factors on vegetation coverage (Lu, Yu, Liu, & Dhruba, 2015; Zhou et al., 2015).

Different contribution assessment methods were used to distinguish the contribution of human activity and climate factors to vegetation growth (Yang et al., 2018). Using the residual method, Tong, Zeng, and Wang (2016) found both climate change and human activities were important factors on vegetation variation in Shanxi Province, but the influence of human activities was relatively higher. Using correlation analysis, climate change only had a marginal effect on vegetation greenness in a typical hilly–gully basin on Loess Plateau, with a contribution of 9.3% (Bai, Mo, Liu, & Hu, 2019). At a larger scale, the contribution of human activities may also surpass the impact of climate change. According to Li, Peng, and Li (2017) and Gang et al. (2018), human activities accounted for 55% and 78.45% of vegetation changes, respectively, over 2000–2015 on Loess Plateau. However, Zheng et al. (2019) distinguished that the contribution of climate change and human activities to grassland variation on Loess Plateau was 57.65% and 42.35% during 2000 to 2016. The determinants of vegetation coverage are complicated, containing numerous impact factors which may be ignored. Besides that, the correlation coefficients of variables may also exist bias, big, or small. In this case, the reliability of the contribution cannot be guaranteed. As a result, we only compare the standard coefficients of different impact factors to judge the relative impact degree on vegetation change.

Under the joint effects of ecological protection policies, rural labor transfer, and climate change, how would the vegetation coverage change? To what extent would these factors affect it? Although quantities studies have discussed on this topic, rare studies combined the labor transfer with ecological policies and climate change.

NDVI (Normal Difference Vegetation Index, NDVI) is one of the most commonly used vegetation indexes, which can reflect vegetation coverage at a large spatial–temporal scale (Du, 1999). Thus, it has been widely applied in many studies (Anyamba & Tucker, 2005; Carlson & Ripley, 1997). Some studies show that NDVI is positively related to vegetation coverage in areas with low vegetation coverage (Watinee & Netnapid, 2013), which means NDVI is suitable for representing the vegetation coverage in North China. Therefore, this paper used NDVI to study the vegetation coverage change on Loess Plateau.

In this research, we selected the NKEFA on Loess Plateau as the study area, based on MODIS‐NDVI data between 2000 and 2015, analyzed the spatiotemporal changes in NDVI, and quantitatively explored the impact of climatic factors and human activities on NDVI changes. Besides that, Yanchi County was selected as a case area to carry out a household survey, analyzing the driving mechanism of vegetation change and distinguishing the key factors in ecosystem changes. The study of dynamic vegetation change is helpful to understand the role of ecological protection policy in ecosystem restoration, which can provide some academic support for policy makers in sustainable natural resources utilization and ecosystem conservation.

## 2 MATERIALS AND METHODS

### 2.1 Study area

**Figure 1:**
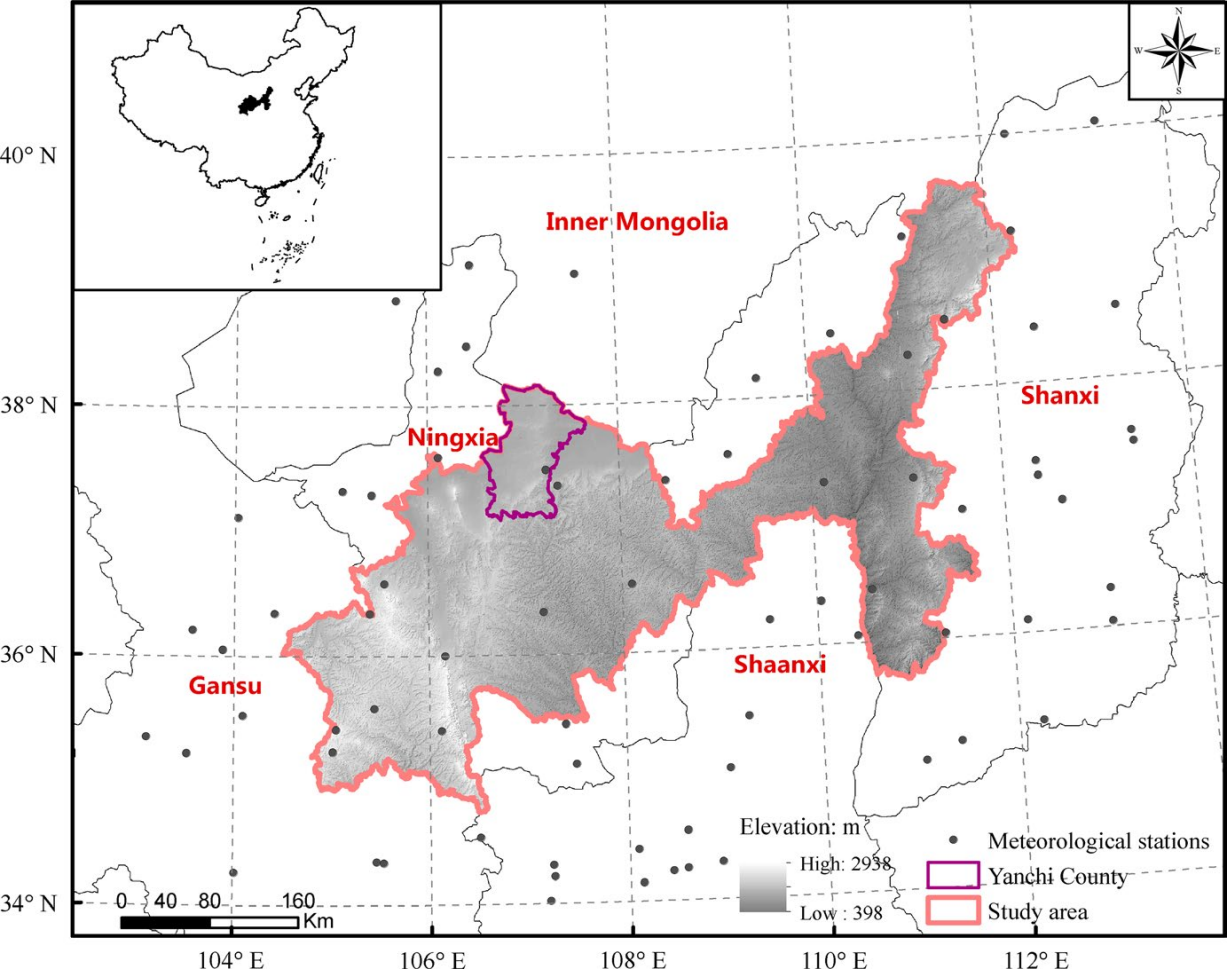
Location of study area and the distribution of meteorological stations

Located in north‐central of China (Figure 1), the NKEFA on Loess Plateau covers 45 counties across Gansu, Ningxia, Shaanxi, and Shanxi, including 112,050.5 km^2^ as a whole. The study area has a typical continental climate, with a mean annual precipitation of 150–700 mm which mostly concentrates in summer. Zonal vegetation includes forest, forest steppe, dry steppe, and desert steppe, showing a zonal change from southeast to northwest, corresponding to the distribution of temperature and precipitation. Influenced by natural conditions, landforms include numerous gullies, thick and loose loess, and severely damaged vegetation cover. As a result, this region is characterized by severe soil erosion and high risk of land degradation.

### 2.2 Data source

#### 2.2.1 NDVI data

The MODIS data (MOD13A3), with a 1‐km spatial resolution and 30‐day intervals, were quality guaranteed after geometric precision correction, radiometric calibration, and atmospheric correction. Considering the climate characteristics and vegetation growth status on Loess Plateau, May to September was selected as the growing season. NDVI of growing season was calculated as the mean value of the monthly NDVI from 2000 to 2015, which was used to represent the average NDVI of the year.

#### 2.2.2 Meteorological data

The mean value of monthly temperature and precipitation data of 77 meteorological stations located in or around the study area were used in this study during 2000 to 2015 (Figure 1). These data were obtained from the Meteorological Data Sharing Service System of the China Meteorological Administration (http://cdc.cma.gov.cn). The meteorological data were interpolated according to the spatial resolution of NDVI data.

#### 2.2.3 Statistical data

Statistical data at county level was used to comprehensively analyze the effects of labor transfer and ecological protection policies on NDVI change. The statistical data was obtained from the statistical yearbook and local survey, including GDP, number of population, farmland area of Yanchi, number of rural workers, number of rural laborers engaged in farming activities, area of newly planted arbor forests and shrub wood with a survival rate exceed 85% and subsidies for afforestation.

**Figure 2:**
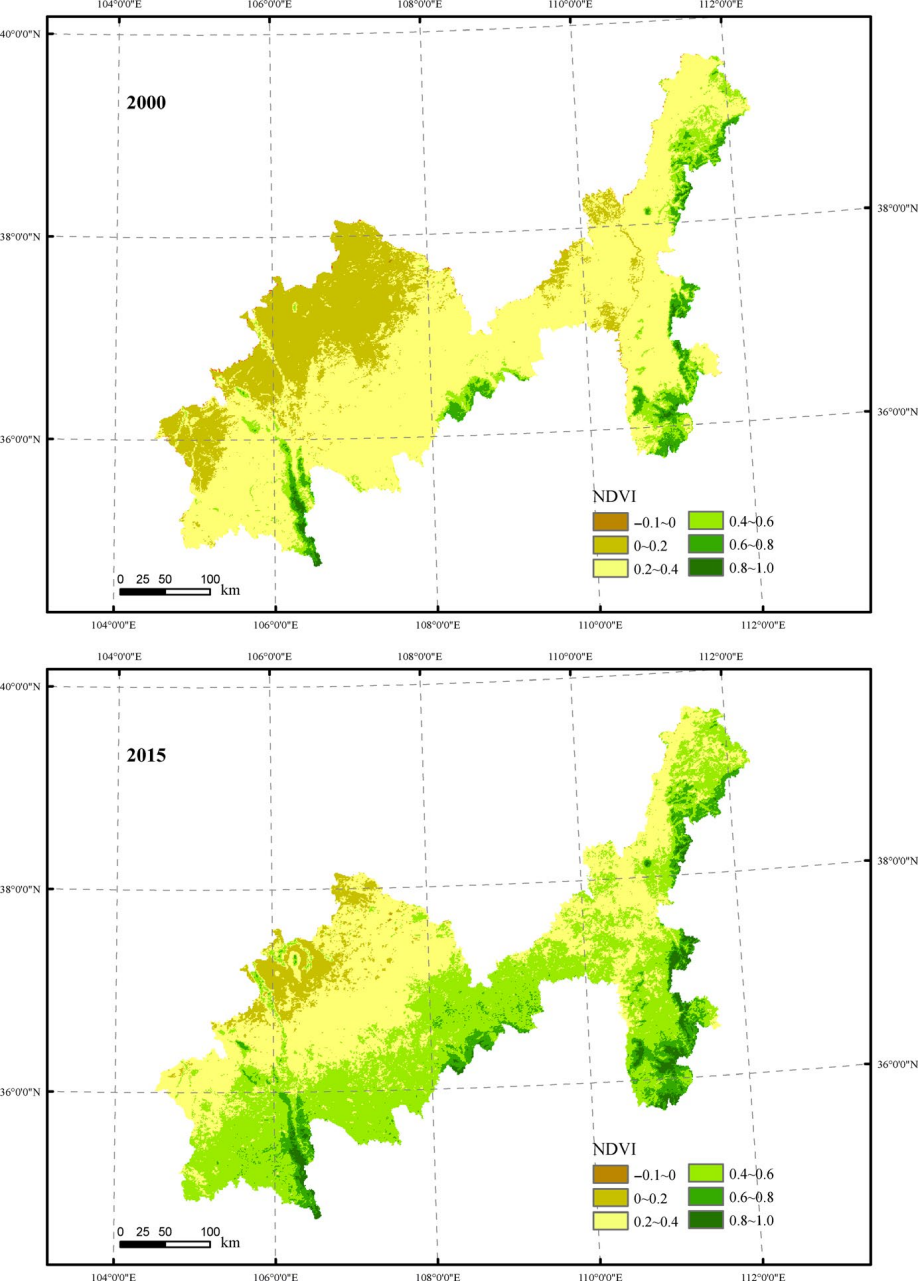
Spatial distribution of NDVI in growing season of 2000 and 2015

#### 2.2.4 Household survey data

Yanchi County was one of the first round counties implemented GFG project in NKEFA on Loess Plateau (Wang, Hao, Zhai, & Liu, 2017), located in the east of Ningxia Province. The area is a typical zone of fragile agro‐pastoral interlaced region, north connecting to Mu Us Sandland and south connecting to Loess Plateau (Zhong et al., 2018). Due to natural conditions and human activities, Yanchi County was faced with various ecological problems during the past decades, such as land desertification, overgrazing in the grassland, vegetation degradation, water and soil erosion, and scarcity of water resources (Wang, Hao, Zhang, Zhai, & Zhang, 2018). As a result, we selected Yanchi County as the case area for household survey, to observe the driving factors of NDVI in NKEFA. Using stratified random sampling method, a survey of 222 households was conducted in 2015. Survey methods include questionnaire investigation, semistructure interviews, and meetings. The questionnaire covered basic information of rural family, labor allocation, livelihood capital, the recognition of ecological compensation, the participation willingness of compensation policy and the expectation to eco‐compensation subsidies, etc.

### 2.3 Methods

#### 2.3.1 Trend analysis

The trend of NDVI change between 2000 and 2015 was calculated as the slope of linear regression. The statistical significance of the trend was assessed by two‐tailed significance tests. The formula is as follows:

(1) $$\text{Slope}=\frac{n\times \sum_{i=1}^{n}i\times {\text{NDVI}}_{i}-\sum_{i=1}^{n}i\times \sum_{i=1}^{n}{\text{NDVI}}_{i}}{n\sum_{i=1}^{n}i^{2}-{\left(\sum_{i=1}^{n}i\right)}^{2}}$$

where *i* is the order of year from 1 to *n*;* n* is the number of study years; and NDVI*_i_* is the value of year *i* which was calculated by monthly NDVI data in the growing season. If slope > 0, the trend of NDVI increased during the study period; otherwise, it decreased.

#### 2.3.2 Partial correlation analysis

Partial correlation analysis is used to study the relationship between two specific variables. When two variables associated with the third variable simultaneously, the impact of the third one is excluded through partial correlation analysis. In this way, only the correlation of the other two variables is estimated. The formula is as followed:

(2) $$r=\frac{\sum_{i=1}^{n}\left({\text{NDVI}}_{i}-\bar{\text{NDVI}}\right)\left(y_{i}-\overset{¯}{y}\right)}{\sqrt{\sum_{i=1}^{n}{\left({\text{NDVI}}_{i}-\bar{\text{NDVI}}\right)}^{2}\times \sum_{i=1}^{n}{\left(y_{i}-\overset{¯}{y}\right)}^{2}}}$$

where *r* is the correlation coefficient; $\bar{\text{NDVI}}$is the mean value of NDVI*_i_* from 2000 to 2015; *y_i_* is the mean temperature or precipitation in growing season of year *i*, $\overset{¯}{y}$ is the mean value of *y_i_* during 2000 to 2015.

The partial correlation coefficient is calculated based on the correlation coefficient, the formula is as followed:

(3) $$r_{ab\cdot c}=\frac{r_{\mathrm{ab}}-r_{\mathrm{ac}}r_{\mathrm{bc}}}{\sqrt{\left(1-r_{\mathrm{ac}}^{2}\right)\left(1-r_{\mathrm{bc}}^{2}\right)}}$$

where $r_{ab\cdot c}$ is the partial correlation coefficient between variables *a* and *b* after fixing variable *c*;* r_ab_*,* r_ac_*,* r_bc_* are the correlation coefficients between variables *a* and *b*,* a* and *c*,* b* and *c*, respectively.

#### 2.3.3 Stepwise regression analysis

In this paper, a stepwise regression statistical model was applied to analyze the determinants of NDVI changes in a case area. The stepwise regression statistical model is a semiautomated process of building a model by successively adding or removing variables. This method introduces the variables that have passed the significance test, which is suitable for the multivariate analysis. The formula is as followed:

(4) $${\text{NDVI}}_{i}=\alpha +\sum \beta_{j}x_{\mathrm{ij}}+\varepsilon$$

where *β_j_* is the parameter of variable *j*;* x_ij_* is the value of variable *j* of the year *i*;* α* is a constant; *ε* is the error term.

In this study, the independent variables were selected to reflect the natural factors, labor force factors, and policy factors (Table 1). Precipitation and temperature were selected to represent the natural factors. GDP per capita, farmland area per labor, and proportion of rural laborers engaged in farming activities were selected as variables which reflect economic pressure on ecosystem. Afforestation area and GFG subsidy funds were selected to reflect the impact of ecological protection policies.

**Table 1:** Model variables associated with NDVI changes

Type	Independent variable	Explain
Natural factors	Precipitation	Mean precipitation in growing season
	Temperature	Mean temperature in growing season
Labor force factors	GDP per capital	GDP/number of population
	Farmland area per labor	Farmland area/number of rural workers
	Proportion of rural laborers engaged in farming activities	Number of rural laborers engaged in farming activities/number of rural workers
Policy factors	Afforestation area	Area of newly planted arbor forests and shrub wood with a survival rate exceed 85%
	GFG project subsidy funds	Including subsidies for afforestation, food subsidies, cash, and living allowance

## 3 RESULTS

### 3.1 Spatial patterns of NDVI

The results showed that NDVI in study area is low in the northwest and high in the southeast, corresponding to the distribution of desert, steppe, and forest. High value of NDVI is mainly distributed in Liupanshan Mountain, Wuling Mountain, and Lvliang Mountain. These regions are covered by broad‐leaved forest, evergreen coniferous forest, and shrub grassland. Low value is mainly distributed in Dingbian, Yanchi, Tongxin, Haiyuan, and Huining County these dry areas, where the mean values of NDVI are generally lower than 0.3 (Figure 2).

### 3.2 Trends of NDVI

**Figure 3:**
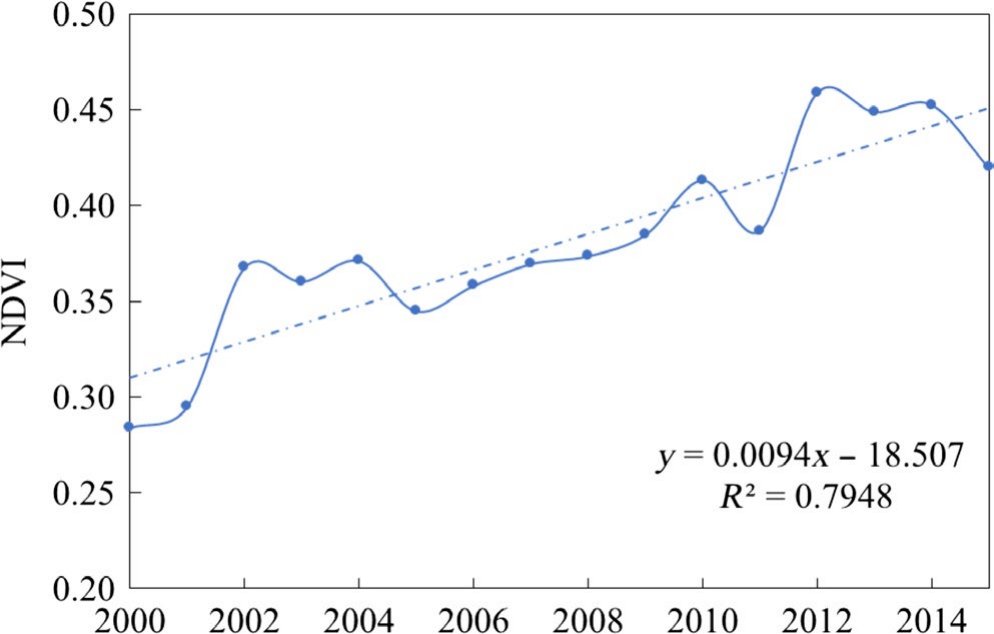
Variations of average NDVI in study area between 2000 and 2015

From 2000 to 2015, NDVI of the study area showed a remarkable increasing trend. The average NDVI in growing season of the region increased by 47.8%, with a rate of 0.085/10a. The vegetation restoration was characterized by three periods, with a fast‐growing period during 2000–2004, a steady increasing period during 2005–2010, and a fluctuation increasing period during 2011–2015 (Figure 3).

**Figure 4:**
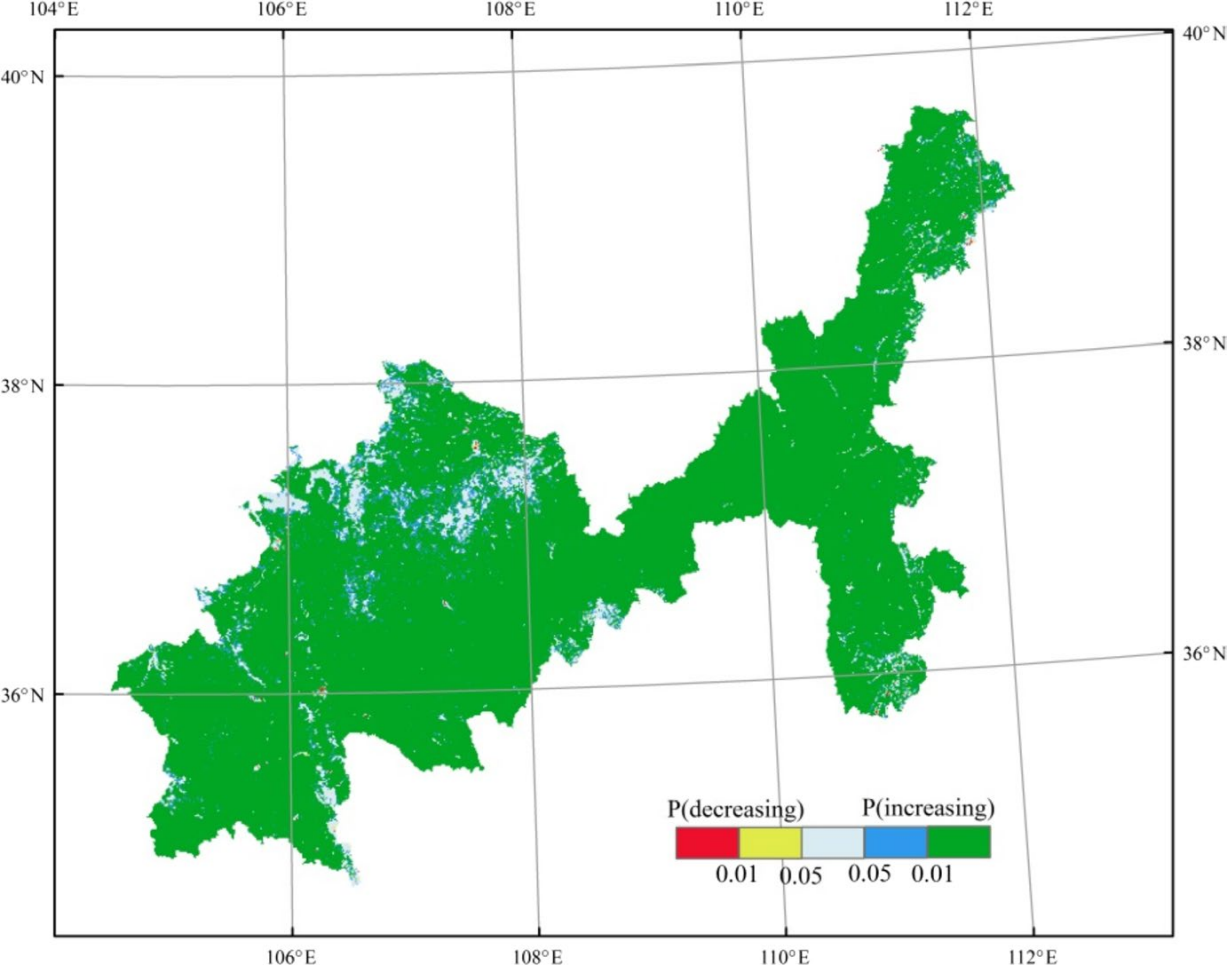
Significance levels of NDVI change between 2000 and 2015. Note: P (decreasing) and P (increasing) represent the P values of the NDVI change, which is divided into five levels: *p* < 0.01, extremely significantly increased; 0.01 < *p*<0.05, significantly increased; *p* > 0.05, no significant variation; 0.01 < *p*<0.05, significantly decreased; and *p* < 0.01, extremely significantly decreased

Under the overall increasing trend of the spatial average NDVI between 2000 and 2015, the temporal change in NDVI in each raster exhibited spatial heterogeneity. About 71.22% of the study area was observed in an extremely significantly increasing trend while 14.79% belonged to significantly increased area. About 13.96% of the study area showed no significant variation, which were distributed sporadically in north Tongxin, south Dingbian, and east Yanchi (Figure 4). Restricted by the hydrothermal condition, the “low in the northwest and high in the southeast” spatial pattern remained unchanged.

### 3.3 Correlation analysis between NDVI and meteorological factors

**Figure 5:**
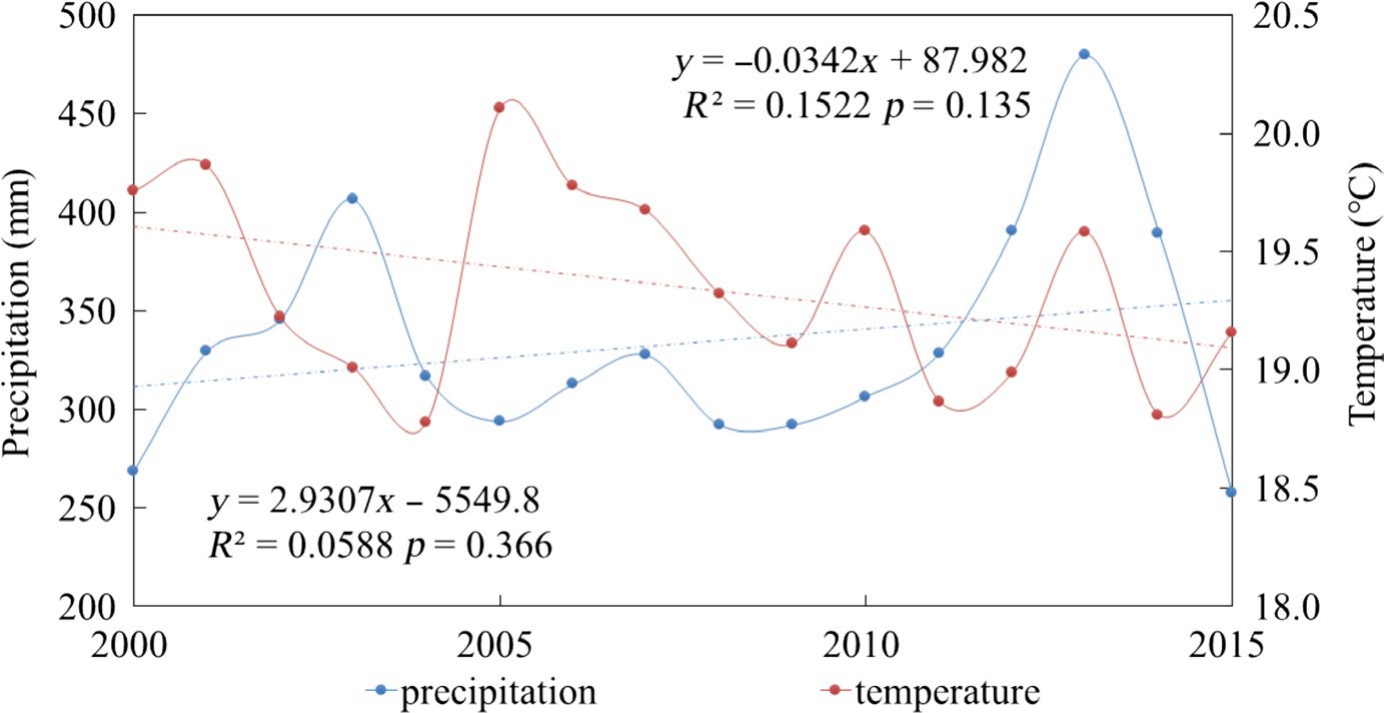
Trend of precipitation and temperature between 2000 and 2015

The average precipitation of study areas showed a weakly increasing trend during 2000 to 2015 at a speed of 29 mm/10a, while the temperature showed a weakly decreasing trend at a speed of 0.34°C/10a. However, due to the drastic fluctuation between different years, the trend of precipitation (*R* ^2 ^= 0.0588, *p* = 0.366) or temperature (*R* ^2 ^= 0.1522, *p* = 0.135) was not significant (Figure 5). Similar results also have been found in the previous research on Loess Plateau (Zhang et al., 2016).

**Figure 6:**
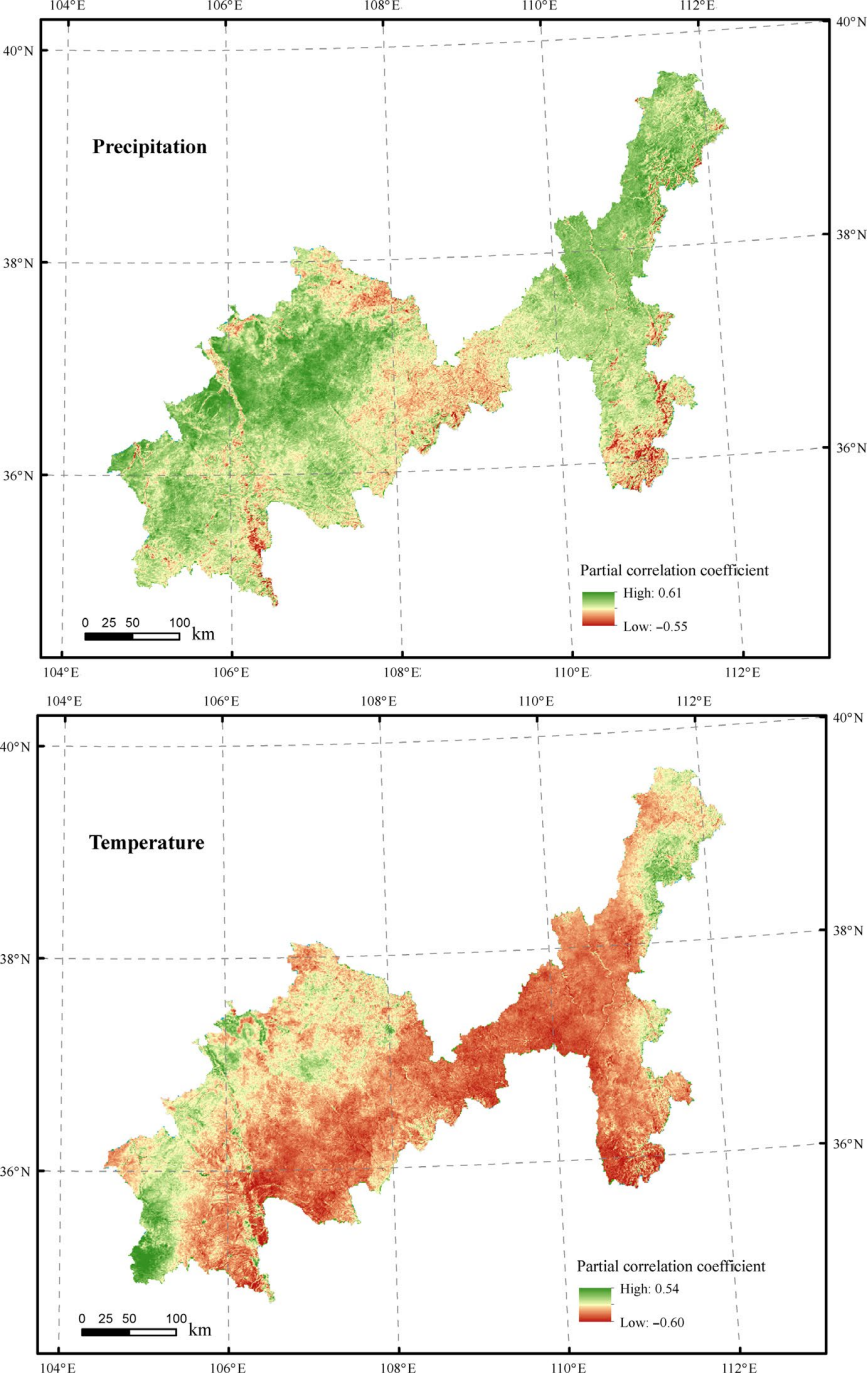
Spatial distribution of partial correlation coefficient between NDVI and temperature, precipitation during 2000–2015

Compared with fluctuant trend of precipitation and temperature, significant increase in vegetation index indicate that climate change is probably not the determinant of the NDVI change. Similar inferences have emerged in previous study (Li et al., 2016). In order to further analyze the response of vegetation coverage to climate change, the partial correlation coefficients between NDVI and meteorological factors of every spatial raster were calculated (Figure 6). The results showed the mean partial correlation coefficients between NDVI and precipitation, temperature were 0.237 and −0.226, respectively. About 95.50% of the study area showed a positive correlation between NDVI and precipitation, especially in low vegetation coverage areas which were distributed in two band regions, including Haiyuan‐Tongxin‐Dingbian‐Yanchi and Pianguan‐Linxian‐Shilou. Among the positive correlation areas, only 0.71% passed *p* < 0.05 test. Similarly, 92.05% of the study area showed a negative correlation between NDVI and temperature, especially in areas with high vegetation coverage, of which only 0.26% passed *p* < 0.05 test.

The above results showed that: (a) During 2000–2015, the spatial average NDVI was positively correlated with precipitation and negatively correlated with temperature, but neither of them showed a statistical significance (*p* > 0.05). (b) The response of vegetation to climate change exhibited large spatial heterogeneity in study area. Areas with low vegetation coverage were more sensitive to temperature changes, while areas with high vegetation coverage were more sensitive to precipitation changes. This suggested that areas with high vegetation coverage had abundant precipitation and thus more susceptible to low temperature; (c) Climate change is not the main factor of NDVI change in study areas between 2000 and 2015.

### 3.4 Relationship between NDVI changes and ecosystem policies

The increase in NDVI was sharp in 2001–2002 and 2011–2012, with the growth rates of 30.23% and 22.03% respectively, which may directly related to the implementation of ecosystem policies.

In August 1999, GFG project started in three provinces including Shaanxi, Gansu, and Sichuan After that, the project extended to 25 provinces, 1897 counties in 2002 (Wang & Chen, 2006), covering the whole study area. From 2000 to 2010, implementation of GFG project in study area had reduced sloping cropland by 1,571 km^2^ and increased ecological land by 1,337 km^2^. Among them, the area of grassland and forest increased by 10.89% and 4.24%, respectively (Lu et al., 2015). This indicates that GFG project had promoted the ecosystem quality, especially in the first round (2001–2002) with powerful policy enforcement and foundation support.

**Figure 7:**
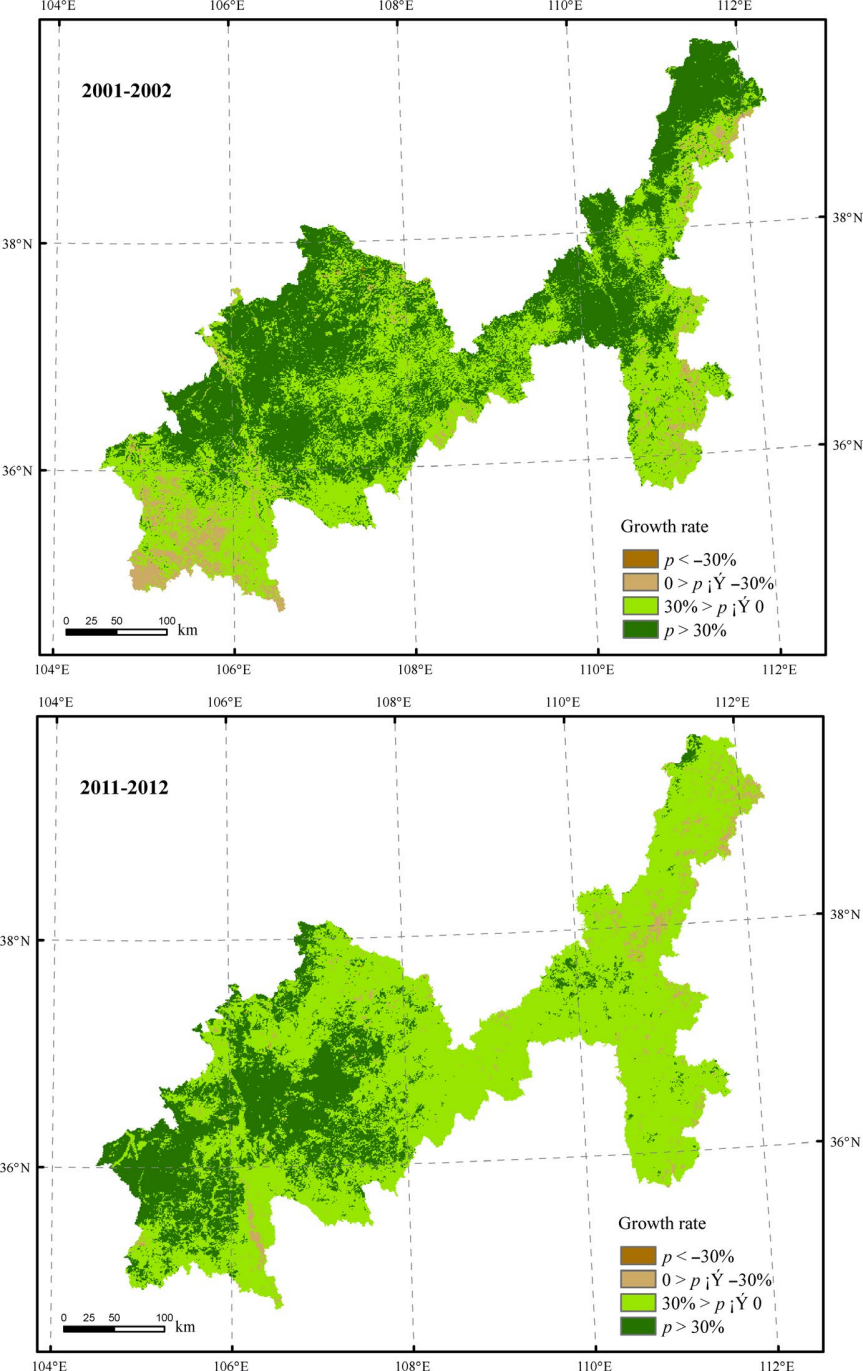
The comparison of vegetation coverage changes between 2001–2002 and 2011–2012

In 2011, the Grassland Subsidy Policy started in 8 major pastoral provinces including Inner Mongolia, Xinjiang, Tibet, Qinghai, Sichuan, Gansu, Ningxia, and Yunnan, and extended to other four semi pastoral provinces in 2012, covering the study area. Refer to an optimal grazing capacity, this policy had rehabilitated grassland eco‐environment. Compared with the vegetation restoration area in the first round (2001–2002), the vegetation was significantly improved in the policy implementing regions during 2011–2012 (Figure 7), including Wuzhong, Guyuan, Haiyuan in Ningxia Province and Qingyang, Huining, Jingning County in Gansu Province.

According to Figure 3, NDVI in the study area increased rapidly after the two policies just implemented. But in the subsequent three years, the NDVI declined with fluctuation, especially in the fourth year (2004–2005, 2014–2015). This may be related to farmer willingness to participate in ecological protection policy. Zhao et al. (2017) also found this trend in their study, but they regard this as a result of low survival rate of trees. With a new round of GFG project officially started, NDVI in study area is expected to rise in the next five years. Maintaining the continuity of ecological protection policy is crucial to ecosystem restoration.

### 3.5 Determinants of NDVI changes in case area

In order to identify the determinants of NDVI changes, this paper selected Yanchi County as a case area to conduct a regression analysis. The average NDVI in Yanchi had increased significantly with a rate of 52.92% from 2000 to 2015. The obvious increase in vegetation coverage in Yanchi has also been confirmed in previous research (Gao et al., 2017). The stepwise regression showed that the proportion of rural laborers engaged in farming activities (*p* < 0.05) and afforestation area (*p* < 0.05) were significant driving factors (Table 2). Proportion of rural laborers engaged in farming activities was negatively correlated with NDVI, indicating that the rural labor transfer had reduced pressure on local ecosystem and promoted vegetation coverage to some extent. Afforestation area was a positive driving factor, indicating that the artificial afforestation had achieved good results. Afforestation area was also used to stand for the effect of ecological protection policies in previous research, with a correlation coefficient of 0.73 between NDVI and it (Zhao et al., 2017). To sum up, labor transfer and the impact of ecological protection policy had surpassed natural factors, becoming the main driving factors of NDVI changes in Yanchi.

**Table 2:** The stepwise regression result of NDVI changes in Yanchi

Model	Standardized coefficients	*t* Test	Sig.
Adjusted *R* ^2^: 0.579 (constant)		9.202	0.000
Proportion of rural laborers engaged in farming activities	−1.057	−4.567	0.001
Afforestation area	0.806	3.482	0.005

Based on the household survey, we found that the government has carried out the GFG project, Grassland Subsidy Policy, Ecological Public‐welfare Forests Protection Policy, and Haba Lake Wetland Compensation Policy. The four policies provided subsidies of 90 yuan/mu, 6 yuan/mu, 11 yuan/mu, and 30 yuan/mu, respectively. All the surveyed households had participated in at least one eco‐compensation policies. Among them, 96.85% of the households were involved in GFG project, 97.30% were involved in Grassland Subsidy Policy, 94.14% had participated in the protection of Ecological Public‐welfare Forest, and 31.08% were involved in wetland subsidies. On average, every household managed 29.78 mu of farmland, 71.30 mu of forest land, and 188.87 mu of grassland. As for policies participation, every household had returned 15.24 mu of farmland to grassland on average. The annual subsidy income of each household was 4,427 yuan. Most farmers believed that the policies were conducive to the local ecosystem protection and the ecological environment had improved significantly, since the frequency of sandstorm had sharply decreased in recent years. About 56.5% of the households were satisfied with the eco‐compensation policies, and 60.8% of the surveyed households would continue to participate in eco‐compensation policies. It showed that the eco‐compensation policies were effective, which led to a win–win for both ecosystem restoration and rural income improvement.

Stepwise regression analysis and household survey showed that the ecological protection policy played an important role in promoting vegetation restoration in Yanchi. As the basic resource users and policy participants, the willingness of farmers will directly affect the results of ecosystem restoration policies (Li et al., 2010). The diversity of farmers’ livelihood is the main factor influencing the farmers’ willingness to participate in eco‐compensation policies. According to the household survey, 58.1% of rural households had nonagricultural employment and 40.8% of rural labors had a short‐term or long‐term nonagricultural employment. Based on indicators such as present way of make a living, major income sources, and the job of major labor force, four household types are identified, including full‐time farming households, farming‐dependent households, nonfarming‐dependent households, and nonfarming households (Zhang, Zhang, Yan, & Wu, 2008). Among the four types, households who were willing to participate in policies took up 53.7%, 53.4%, 54.7%, and 66.7%, respectively. Nonfarming households did not depend on farmland, while full‐time farming households had a higher dependence on land, whose income was mainly from agricultural or sheep products. Other relative studies also confirmed that livelihood diversification could effectively reduce their reliance on natural resources, which lead to a higher willingness to participate in eco‐compensation policies (Li & Cai, 2014; Wang, 2010).

Meanwhile, 35.59% of the surveyed households were dissatisfied with the compensation standards and refused to participate in eco‐compensation policies. Firstly, the compensation subsidies were much lower than the income of animal husbandry. Secondly, the policies lacked stability, such as the Wetland Protection Policy, with a compensation standard reduced from 60 yuan/mu in 2014 to 30 yuan/mu in 2015. In order to improve the farmers' willingness to participate in ecological protection policies, the variety of livelihood and the stability of policy should be considered when making eco‐compensation policies.

## 4 CONCLUSION AND DISCUSSION

1. Since 1999, China has carried out a series of ecological protection policies including GFG project, Grassland Subsidy Policy, and Ecological Public‐Welfare Forest Protection Policy. As one of the main implementing regions of these policies, NDVI of NKEFA in the Loess Plateau increased remarkably with a rate of 0.085/10a.
2. As the new round of GFG project has officially started, the vegetation coverage in study area is expected to keep rising generally. The participation willingness of farmers will determine the effect and sustainability of ecological protection policies. According to the household survey in Yanchi, the livelihood types of farmers, compensation standard, and policy stability will affect farmer willingness. Given the importance of the NKEFA, it is necessary to increase the ecological compensation intensity and maintain the continuity of ecological compensation policy. In order to achieve a win–win situation of farmers' livelihood and ecosystem conservation, a targeted ecological compensation policy should be considered.
3. In this paper, NDVI was used as the index of ecosystem restoration, which took more consideration of its ecosystem services function in sand fixation and soil conservation. However, as the largest arid and semiarid zone in China, some researchers considered that large scale of vegetation restoration was not suitable for Loess Plateau due to a lack of water resources (Jin et al., 2018).In the future study, we should pay more attention to the comprehensive influence of ecological protection policy on ecosystem services at regional scale. Researches have already found that there is a trade‐off between different types of ecosystem services (Dai, Wang, & Zhu, 2015; Rao, Lin, Wang, Zhang, & Lu, 2015). The impact of ecological protection policy on different ecosystem services is also different. According to a study in the Loess Plateau (Feng et al., 2016), the vegetation productivity was enhanced by the ecological policies at the cost of river runoff reduction. Therefore, in the future research, analyzing the trend of different ecosystem services based on regional ecological function positioning is necessary for promoting the scientific implementation and sustainable management of ecological protection policies.

## CONFLICTS OF INTEREST

The authors declare no conflict of interest.

## AUTHOR CONTRIBUTION

Haiguang Hao and Ruixue Zhai designed the study, analyzed the data, and wrote the manuscript; Yuanyuan Li, Huiyuan Zhang, and Haiyan Liu contributed to data collection and manuscript revision.

## Data Availability

Data on changes in vegetation cover, temperature, and precipitation are available from datadryad (http://doi.org/10.5061/dryad.rj2gf5j).
